# *Achyranthis radix* Extract Enhances Antioxidant Effect of Placenta-Derived Mesenchymal Stem Cell on Injured Human Ocular Cells

**DOI:** 10.3390/cells13141229

**Published:** 2024-07-21

**Authors:** Dae-Hyun Lee, Ji Woong Han, Hyeri Park, Se Jin Hong, Chan-Sik Kim, Young Sook Kim, Ik Soo Lee, Gi Jin Kim

**Affiliations:** 1 Department of Biomedical Science, CHA University, Seongnam 13488, Republic of Korea; ldh1532@chauniv.ac.kr (D.-H.L.); hyeyeyeri@chauniv.ac.kr (H.P.); indy14@chauniv.ac.kr (S.J.H.); 2 Advanced PLAB, PLABiologics Co., Ltd., Seongnam 13522, Republic of Korea; jwhan@plabiologics.com; 3 Korean Medicine Convergence Research Division, Korea Institute of Oriental Medicine, Daejeon 34054, Republic of Korea; chskim@kiom.re.kr (C.-S.K.); ykim@kiom.re.kr (Y.S.K.)

**Keywords:** ocular diseases, macular degeneration, placenta-derived mesenchymal stem cells, *Achyranthis radix* extract, antioxidants, retinal injured model, retinal pigment epithelial cell, regeneration

## Abstract

Age-related ocular diseases such as age-related macular degeneration, glaucoma, and diabetic retinopathy are major causes of irreversible vision impairment in the elderly. Conventional treatments focus on symptom relief and disease slowdown, often involving surgery, but fall short of providing a cure, leading to substantial vision loss. Regenerative medicine, particularly mesenchymal stem cells (MSCs), holds promise for ocular disease treatment. This study investigates the synergistic potential of combining placenta-derived MSCs (PD-MSCs) with *Achyranthis radix* extract (ARE) from *Achyranthes japonica* to enhance therapeutic outcomes. In a 24-h treatment, ARE significantly increased the proliferative capacity of PD-MSCs and delayed their senescence (* *p* < 0.05). ARE also enhanced antioxidant capabilities and increased the expression of regeneration-associated genes in an in vitro injured model using chemical damages on human retinal pigment epithelial cell line (ARPE-19) (* *p* < 0.05). These results suggest that ARE-primed PD-MSC have the capability to enhance the activation of genes associated with regeneration in the injured eye via increasing antioxidant properties. Taken together, these findings support the conclusion that ARE-primed PD-MSC may serve as an enhanced source for stem cell-based therapy in ocular diseases.

## 1. Introduction

Age-related ocular diseases have emerged as a significant global health concern, particularly with the increasing aging population and the rising prevalence of diabetes worldwide [1]. The demand for treating posterior segment ocular diseases like age-related macular degeneration (AMD), glaucoma, and diabetic retinopathy (DR) is on the rise, severely impacting the quality of life for patients [2]. AMD, a primary cause of irreversible blindness in the elderly, along with conditions like glaucoma and DR, significantly contribute to visual impairment in older adults [3]. Glaucoma is an irreversible neurodegeneration that involves retinal nerve fiber layer thinning, optic nerve head cupping, and retinal ganglion cell (RGC) death [4], while DR originates from damage to retinal blood vessels as well as neurodegeneration of the retina [5]. RGC death is also one of the earliest signs of diabetic retinal neuropathy, and diabetic optic nerve damage even precedes retinal abnormality [6].

The leading theory of aging, the free radical theory, highlights oxidative stress, particularly from accumulated reactive oxygen species (ROS), as a key contributor to age-related ocular diseases. Oxidative stress plays a crucial role in the pathological processes of various retinal dysfunctions. The anatomical features of the eye render the retina particularly vulnerable to ROS production, a susceptibility that tends to increase with age [7]. The retina, especially the photoreceptors and retinal pigment epithelium, is rich in polyunsaturated fatty acids, making it prone to lipid peroxidation. The persistence of prooxidant factors and a decline in antioxidant capacity with age may expedite oxidative stress. Consequently, elevated levels of ROS and intracellular Ca^2+^ concentrations represent common pathological changes in the retina. Numerous studies have suggested that oxidative stress injury serves as the initial trigger for the cell death of retinal neurons. Ocular diseases often share similar cellular mechanisms [8]. This imbalance leads to structural and functional impairments in the eyes, such as vascular issues and neovascularization. Pathological changes affect various eye components, resulting in surface inflammation, lens opacity, increased intraocular pressure, and retinal degeneration [9]. While local or systemic antioxidant therapy has garnered interest, ocular structural complexities often lead to systemic side effects [7]. To achieve long-term disease control with minimal side effects, alternative therapies like natural antioxidants and cell-based approaches are being explored for preventing and treating age-related eye diseases.

*Achyranthes japonica*, a natural product, is classified as a perennial herb of the genus *Achyranthes*. The roots of *A. japonica*, sometimes referred to as *Achyranthis radix*, have been employed in traditional medicine for the treatment of arthritis, menstrual disorders, and edema [10,11]. The pharmacological activities of *Achyranthis radix* extract (ARE) have been extensively studied through experimental research [12]. These activities include anti-tumor, immunostimulatory, uterotonic, anti-fertility, cognition-enhancing, anti-bacterial, anti-aging, anti-inflammatory, and anti-osteoporotic effects [13].

Regenerative medicine has made notable advancements, with mesenchymal stem cells (MSCs) emerging as a promising therapeutic option due to their anti-inflammatory properties, antioxidant effects, ability to promote tissue repair, and immune regulatory effects [14]. Extensive research on both humans and animals highlights the potential of MSCs from diverse sources like bone marrow (BM), adipose tissue (AD), the amniotic membrane (AM), and the umbilical cord (UC) in treating ocular diseases [15]. Retinal diseases in the posterior eye segment, including AMD, DR, uveitis, and retinal vasculitis, exhibit pathological angiogenesis and damage to retinal cells, ultimately resulting in permanent vision loss [16]. Several studies have demonstrated that MSCs or MSC-conditioned media containing small molecules and various neurotrophic factors can induce MSC differentiation towards retinal cells and eye field neuroectoderm [17,18]. While MSCs have demonstrated the ability to differentiate into retinal cell types in vitro, it remains uncertain whether these differentiated cells can function effectively in vivo [19]. Notably, subretinal transplantation of human embryonic stem cell-derived retinal pigment epithelium (RPE) cells showed tolerance in AMD and Stargardt’s disease patients [20,21]. Additionally, autologous transplantation of human induced pluripotent stem cell-derived RPE cells did not result in significant clinical improvement in tested AMD patients [22]. Conversely, MSCs promote retinal layer repair and regeneration through their paracrine factor secretion of neurotropic factors for repair of neuro-retinal cells. Additionally, MSCs exhibit immunomodulatory capabilities, effectively attenuating the pro-inflammatory conditions typically associated with retinal degenerative diseases. Furthermore, they secrete anti-angiogenic factors, acting to impede the pro-angiogenesis implicated in the development of specific ocular diseases [23]. The promising results observed in injecting MSCs in animal models of retinal degeneration prompted the commencement of several clinical trials. However, the outcomes from these trials have been diverse, raising numerous questions about the clinical application of MSCs. While some cases have shown improved visual acuity [24], others have faced complications such as fibrosis and retinal detachment [25,26]. The variable results emphasize the need for further investigation and careful consideration before the clinical use of MSCs in treating retinal degenerative conditions can become widespread. MSCs have been utilized and have demonstrated effective efficacy in laboratory studies [27,28]. However, understanding the intricate relationship between various immunogenic environments and their impact on MSC-derived therapeutic effects requires a more profound exploration at the molecular level.

Human placenta-derived mesenchymal stem cells (PD-MSCs), sourced from fetal tissue, possess several notable advantages within the MSC category [29]. These advantages include robust immunosuppressive capabilities and a heightened proliferative potential compared to other MSCs [30]. Consequently, PD-MSCs require fewer passages to achieve a substantial cell count, thereby minimizing the risk of cellular senescence. MSCs derived from placenta are believed to be more primitive than cells obtained from adult tissues. Moreover, placenta is plentiful and usually discarded as a recyclable biological waste. Previously, we reported that PD-MSCs functionally enhanced by pigment epithelium-derived factor (PEDF) overexpression, PD-MSCs^PEDF^, showed their potential regenerative effects on RPE cells damaged by hydrogen peroxide (H_2_O_2_)-induced oxidative stress [31] and restored the visual cycle through a mitophagic mechanism in RPE cells in hydrogen peroxide (H_2_O_2_)-injured rat retinas [32]. These data suggest that priming PD-MSCs with PEDF might be a new cell therapy for the treatment of retinal degenerative diseases. In addition, recent investigations have focused on novel strategies for augmenting the functionality of MSCs. These include techniques such as cell priming and genetic changes aimed at improving the efficiency of transplantation, with the ultimate goal of raising the therapeutic efficacy of stem cell-based treatments [31,33]. Despite MSCs’ potential for treating ocular diseases due to their secretion of soluble factors and modulating effects, challenges include poor cell survivability and self-renewal post-transplantation [34]. The ongoing maintenance and enhancement of the viability and excretion of MSCs continue to present significant challenges within this domain, necessitating additional research [35]. In recent years, there has been an exploration of priming approaches to enhance the function of MSC, leading to the creation of cellular products with enhanced potential for various clinical applications [36]. The utilization of priming, involving pharmacological or chemical agents, stands as a promising strategy to bolster MSC engraftment and survival in damaged tissues, ultimately enhancing therapeutic efficacy [37]. *Achyranthis radix* is a medicinal herb known for its anti-inflammatory properties, and our recent study has shown that its extract (ARE) has therapeutic effects on the inflammatory condition of dry eye disease. This suggests that ARE is a promising natural material for research on ocular diseases [38]. Nevertheless, the precise synergistic impact of the combination of PD-MSCs and ARE on ocular diseases remains unclear. Hence, the aim of this study was to examine the impact of ARE on human PD-MSCs, with the goal of augmenting the therapeutic capabilities of these cells for the management of ocular diseases.

## 2. Materials and Methods

### 2.1. Preparation of Achyranthis radix Extract (ARE)

*Achyranthis radix* was purchased from Kwangmyungdang Medicinal Herbs, Ulsan, Republic of Korea. The voucher specimen (KIOM-USL) has been deposited in the Herbarium of the Korea Institute of Oriental Medicine (KIOM, Daejeon, Republic of Korea). *Achyranthis radix* (1 kg) was extracted with distilled water (10 L) at 100 °C for 3 h using a heat-reflux extractor. The extract solution was filtered and evaporated under reduced pressure using a rotary evaporator (N-1200A, Eyela, Tokyo, Japan) at 50 °C. The concentrated extract solution was then freeze-dried using a freeze-dryer (FDU-2100, Eyela, Tokyo, Japan) at −80 °C for three days to obtain a dried extract powder (298.5 g, yield 29.85%). 

### 2.2. HPLC Analysis of ARE

The purity of all three reference standards of ecdysterone, 25R-inokosterone, and 25S-inokosterone (ChemFaces, Wuhan, China) was >98%. HPLC-grade of acetonitrile, methanol, and water were purchased from J.T. Baker, Phillipsburg, NJ. Analytical-grade formic acid was purchased from Merck, Darmstadt, Germany. ARE (20 mg) was dissolved in HPLC-grade methanol (10 mL), and the solution was filtered using a 0.45-μm syringe filter (Whatman, Clifton, NJ, USA) before HPLC analysis. Standard stock solutions of three reference standards (all at 1 mg/mL) were prepared in HPLC-grade methanol, stored at <4 °C. HPLC analysis was conducted with a 1290 Infinity II LC Systems (Agilent Technologies, Santa Clara, CA, USA) equipped with a binary pump, vacuum degasser, autosampler, column compartment, and diode array detector (DAD). Chromatographic separation was performed using an ACQUITY UPLC BEH C18 column (100 × 2.1 mm, 1.7 µm, Waters Corporation, Milford, MA, USA) and the column temperature was maintained at 35 °C. The mobile phase consisted of acetonitrile (A) and 0.1% formic acid in water (B) with gradient elution. The gradient solvent system was optimized as follows: 90% B (0–5 min), 90–80% B (5–20 min), and 80% B (20–25 min). The column was re-equilibrated with 90% B for 5 min before each analysis. The flow rate was set at 0.25 mL/min, and the injection volume for each sample was 3.0 μL. The detection was performed at 245 nm. Data were collected and analyzed using the Agilent ChemStation software C.01.10 (Agilent Technologies).

### 2.3. Cell Culture

Placentas obtained at term (37 gestational weeks) in women who did not experience any obstetric, perinatal, or surgical problems were used. The IRB at CHA General Hospital, Seoul, Republic of Korea, approved the sample collection and usage for the study (IRB 07-18). Written informed consent was obtained from all women who participated. Placenta-derived mesenchymal stem cells (PD-MSCs) were isolated using a previously described method [39]. Alpha-modified minimal essential medium (α-MEM, HyClone, Logan, UT, USA) was used to maintain PD-MSCs. This medium was supplemented with 10% fetal bovine serum (FBS, Gibco, Carlsbad, CA, USA), 1% penicillin/streptomycin (P/S, Gibco), and 25 ng/mL human fibroblast growth factor-4 (hFGF-4, PeproTech, Rocky Hill, NJ, USA). The cells were maintained at 37 °C in a humid atmosphere containing 5% CO_2_ for 24 h. PD-MSCs were subjected to treatment with concentration of 13.5 and 135 ng/mL of ARE for a duration of 24 h. 

### 2.4. In Vitro Coculture System

Human retinal pigment epithelium-derived ARPE-19 (ATCC, Manassas, VA, USA) was maintained in Dulbecco’s modified Eagle medium (DMEM, Gibco) containing 10% FBS (Gibco) and 1% P/S (Gibco). To analyze the effect of naïve PD-MSC or ARE-primed PD-MSC, 1 × 10^5^ of ARPE19 cells were cultured in 6-well plate (Corning, Corning, NY, USA) at 5% CO_2_ and 37 °C. And ARPE19 cells were treated with hydrogen peroxide (H_2_O_2_, 200 μM, Sigma-Aldrich, St. Louis, MO, USA) for 2 h and cocultured with naïve PD-MSC or dose-dependent ARE-primed PD-MSC (5 × 10^3^/cm^2^) in 8-μm pore Transwell^®^ inserts (Corning) in α-MEM (HyClone) containing 1% P/S (Gibco) for 24 h at 5% CO_2_ and 37 °C.

### 2.5. Cell Culture Immunophenotypes by Flow Cytometry

For analysis of the immunophenotyping of cell surface antigens, PD-MSCs were treated with concentrations of 13.5 and 135 ng/mL of ARE and stained with phycoerythrin (PE)-conjugated HLA-DR, THY1 (CD90), and ENG (CD105) antibodies (BD Bioscience, Franklin Lakes, NJ, USA) and fluorescein isothiocyanate (FITC)-conjugated HLA-ABC, CD34, PTPRC (CD45) antibodies (BD Bioscience), using a CytoFLEX flow cytometer (Beckman Coulter, Brea, CA, USA). All data sets were collected from 10,000 cells.

### 2.6. Multilineage Differentiation

To confirm the mesodermal differentiation potential of naïve PD-MSCs and ARE-treated PD-MSCs, cells were seeded in each plate (5 × 10^3^ cells/cm^2^) with each differentiation induction medium for ~21 days using the StemPro Adipogenesis, Osteogenesis Differentiation Kit (Gibco) and Chondrogenic differentiation kit (PromoCell, Heidelberg, Germany) according to the manufacturer’s instructions. After 21 days, the cells were fixed in 4% paraformaldehyde and stained using Oil Red O (Sigma-Aldrich) for lipid droplet identification; the cells were stained using Alcian blue (Sigma-Aldrich), and von Kossa (Sigma-Aldrich) staining with 5% silver nitrate was used to visualize calcium deposits. Each experiment was performed in triplicate. The image data quantification involved the identification of a region of interest (ROI) for the particular cells in each image, accomplished through the use of the selection (freedom) tool in Image J software (Rasband, W.S., ImageJ, U. S. National Institutes of Health, Bethesda, Maryland, USA, https://imagej.nih.gov/ij/, accessed on 12 July 2024, 1997–2024). Subsequently, both the area and mean gray value were measured to compute the integrated density of the inverted selected images. This process allowed for the quantification of positive signals.

### 2.7. Quantitative Real-Time PCR (qRT-PCR)

Total RNA was isolated using TRIzol^TM^ LS reagent (Invitrogen, Carlsbad, CA, USA) according to the manufacturer’s protocol. cDNA was synthesized by reverse transcriptase (RT) from total RNA (500 ng) using SuperScript III RT (Invitrogen). qRT-PCR was performed with primers (Table 1) and SYBR Green PCR Master Mix (Roche, Penzberg, Germany) in the CFX Connect™ Real-Time System (Bio-Rad Laboratories, Hercules, CA, USA). The expression of each gene was quantified using the 2^−∆∆CT^ method. All reactions were performed in triplicate. Each sample was examined in triplicate, with human GAPDH as the internal control for standardization.

### 2.8. Western Blot

The samples were lysed in lysis buffer (Sigma-Aldrich) with a phosphatase inhibitor (AG Scientific, San Diego, CA, USA) and a protease inhibitor cocktail (Roche). Protein lysates were separated by sodium dodecyl sulfate-polyacrylamide gel electrophoresis (SDS-PAGE) and transferred to PVDF membranes (Bio-Rad). Primary antibodies were used as follows: anti-HMOX1 (1:1000, NBP1-97507, Novus Biologicals, Centennial, CO, USA) [40], anti-SOD1 (1:1000, 4266S, Cell Signaling Technology, Danvers, MA, USA) [41], anti-TGFB1 (1:1000, ab92486, Abcam, Cambridge, UK) [42], anti-IL6 (1:1000, ab6672, Abcam) [43]), anti-RPE65 (1:2000, MA1-16578, Invitrogen) [44], anti-RDN11 (1:1000, bs-6214R, Bioss, Woburn, MA, USA), anti-total AKT (1:1000, 9272S, Cell Signaling Technology) [45], anti-phospho AKT (1:1000, 9271S, Cell Signaling Technology) [46], anti-PI3K (1:2000, 4255S, Cell Signaling Technology) [47], and anti-GAPDH (1:3000, LF-PA0018, AbFrontier, Seoul, Republic of Korea) [48]. The following secondary antibodies were used: anti-HRP-conjugated mouse IgG (1:5000, 7076S, Cell Signaling Technology) [49] and anti-HRP-conjugated rabbit IgG (1:5000, 7074S, Cell Signaling Technology) [50]. Each band was subject to chemiluminescent detection using ECL reagent (Bio-Rad) and quantified using Image J software (NIH, Bethesda, MD, USA).

### 2.9. Population Doubling Time (PDT)

To analyze the doubling time, ARE-primed PD-MSCs (passage 6) were seeded in plates at a density of 5 × 10^4^ cells/cm^2^, and the doubling time (DT) of the harvested cells was calculated using the following online algorithm (http://www.doubling-time.com, accessed on 21 May 2024): DT = *t* × log_2_/(logN1 − logN0), where N0 is the number of cells inoculated, N1 is the number of cells harvested, and *t* is the culture time in hours. The experiment was performed at least in triplicate.

### 2.10. ROS Measurement

After the cells were harvested, they were washed in 1 × phosphate-buffered saline (PBS; eLbio). Then, the cells were incubated with 1.5 μM MitoSOX^TM^ (Superoxide staining, red signals; Invitrogen Corporation) and 50 nM MitoTracker^TM^ (Mitochondria staining, green signals; Invitrogen Corporation) for 40 min at 37 °C. After staining, the cells were washed and mounted by using VECTASHIELD^®^ antifade mounting medium with DAPI (Vector Labs, Burlingame, CA, USA). The cells were observed via confocal microscopy (Zeiss 780; Zeiss, Oberkochen, Germany) at 200× magnification, and images of randomized areas of all the slides were captured. We quantified the 29~35 cells were counted (6~7 cells with 5 images).

### 2.11. SA-β-Gal Staining

The senescence activity of each cell type (passage 10) was detected using an SA-β-gal kit (Cell Signaling) according to the manufacturer’s instructions. Cells were fixed for 10 min in a 1× fixative solution at room temperature and then incubated overnight at 37 °C with a 1× SA-β-gal staining solution (pH 6.0). The percentage of SA-β-gal-positive cells among each cell type was analyzed by the program ImageJ (NIH). We quantified the signals from 4~6 cells for each image out of 3 images (14~16 cells). 

### 2.12. Statistical Analysis

Each experiment was carried out in two or three copies. The data are expressed as the mean ± standard error of the mean (SEM). Tukey’s post hoc test was used after one-way ANOVA was used for between-group comparisons of the data obtained at various time points. PRISM 5.01 (GraphPad Software version 5.01, San Diego, CA, USA) was used to evaluate the data, and the *p*-value less than 0.05 was considered statistically significant.

## 3. Results

### 3.1. HPLC Analysis of Compounds in ARE

High-performance liquid chromatography (HPLC) using ultraviolet (UV) detection technique was applied to analyze the main components of ARE, which was performed according to the previously described procedures with slight modifications [51]. 

**Figure 1:**
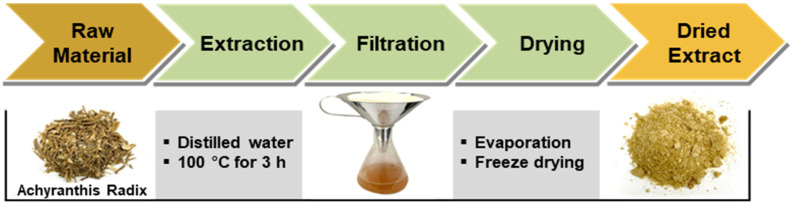
The process of obtaining a dried extract powder from *Achyranthis radix*.

**Figure 2:**
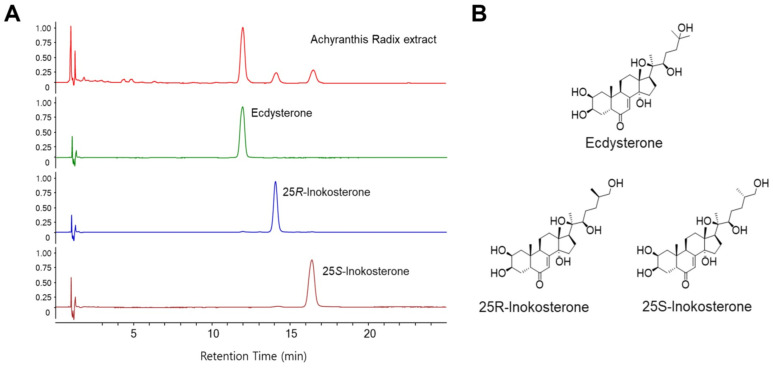
Extraction process of *Achyranthis radix* and HPLC analysis of ARE. (**A**) HPLC chromatograms of ARE and three reference standards (ecdysterone, 25*R*-inokosterone, and 25*S*-inokosterone). Chromatographic conditions are described in the text. Detection was at 250 nm. (**B**) Chemical structures of three standard compounds.

Under the chromatography conditions, three major chromatographic peaks (peaks 1–3) were successfully separated and appeared at retention times of 12.0, 14.2, and 16.5 min, respectively. The process of obtaining the ARE is shown in Figure 1, and HPLC chromatograms of the ARE and three reference standards (ecdysterone, 25R-inokosterone, and 25S-inokosterone) are shown in Figure 2A. Comparison of the UV spectra and retention times of three main peaks in ARE with those of the corresponding reference standards confirmed the identifications of peaks 1 to 3 of ARE as ecdysterone, 25R-inokosterone, and 25S-inokosterone, respectively. The chemical structures of these compounds are presented in Figure 2B. The developed HPLC method was applied to simultaneous quantitative analysis of three main compounds in the ARE. Samples were analyzed in triplicate, the contents of the three compounds in the ARE were in the range 0.25–0.98 mg/g; the most abundant component in the ARE was ecdysterone (0.98 ± 0.05 mg/g), followed by 25*S*-inokosterone (0.34 ± 0.03 mg/g) and 25*R*-inokosterone (0.25 ± 0.02 mg/g).

### 3.2. ARE Enhanced Function of PD-MSC 

**Figure 3:**
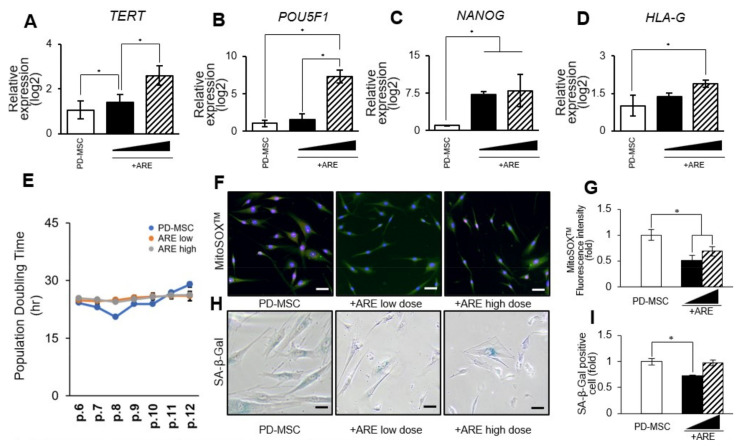

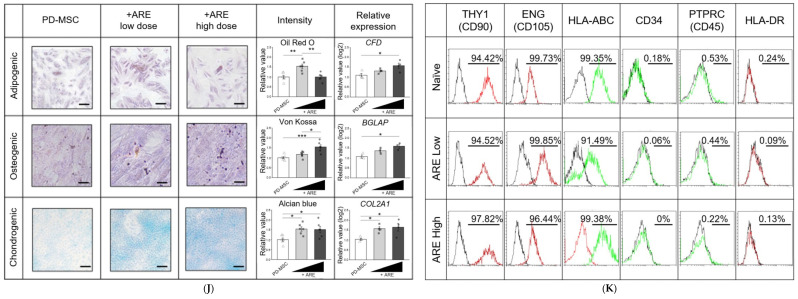
Characteristics of ARE-primed PD-MSC. The mRNA expression of (**A**) *TERT*, (**B**) *POU5F1*, (**C**) *NANOG*, (**D**) *HLA-G* analyzed by qRT-PCR. (**E**) The comparison of doubling time between PD-MSC, ARE low (13.5 ng/mL), and ARE high (135 ng/mL). (**F**) Oxidative stress levels evaluated in PD-MSC, ARE low, ARE high cell using MitoSOX^TM^/MitoTracker staining. (**G**) MitoSOX^TM^ intensity quantified by image J. (**H**) Senescence levels evaluated in PD-MSC, ARE low, ARE high cell using SA-β-Gal staining. (**I**) SA-β-Gal staining intensity quantified by image J. (**J**) Adipogenic, Osteogenic, Chondrogenic differentiation were confirmed by Oil-Red O staining, von Kossa staining, and Alcian blue staining. The staining intensity was quantified by image J. The white bar, gray bar, black bar means PD-MSCs only, PD-MSCs treated with ARE low, PD-MSCs treated with ARE high. The mRNA expression of CFD, BGLAP, and COL2A1 analyzed by qRT-PCR. (**K**) Expression of surface markers for immunomodulation in PD-MSC, ARE low, ARE high. Scale bar = 100 μm (**F**,**H**). Scale bar = 100 μm, (**J**). Each experiment was replicated three times, resulting in a total sample size of three (*n* = 3). In figure (**A**–**I**) White bar, black bar, hatch bar means PD-MSCs only, PD-MSCs treated with ARE low, PD-MSCs treated with ARE high. The data represent the mean ± SEM. Statistical significance was determined by using one-way ANOVA and Tukey’s post hoc test for the comparison of groups, * *p* < 0.05, ** *p* < 0.01, *** *p* < 0.001.

To investigate the cell proliferative effect of ARE on PD-MSC, the cells were treated for 24 h with ARE extracted via hot water at concentrations of 13.5 (ARE low) or 135 (ARE high) ng/mL. We tested a range of concentrations to ensure the selection of the most effective and non-toxic dose (Figures S1 and S2). The ARE priming increased the expression of stemness markers, TERT, POU5F1, NANOG, and HLA-G in PD-MSC in a dose-dependent manner (Figure 3A–D). Stemness denotes the capacity of these cells to differentiate into diverse cell types, and manipulating this characteristic can profoundly impact their behavior. MSCs with enhanced stemness possess an increased ability to self-renew. 

When compared to untreated cells, ARE treatment maintained the PD-MSC proliferative capacity, as measured by population doubling time with cell numbers at each passage (Figure 3E). It is imperative to conduct meticulous monitoring and rigorous safety assessments to mitigate this potential risk. While heightened stemness may amplify the differentiation potential of MSCs, ensuring that the differentiated cells maintain normal functionality is crucial. The utilization of MitoSOX^TM^, a fluorescent probe for detecting mitochondrial superoxide, revealed that the application of ARE resulted in enhanced antioxidant properties of PD-MSCs (Figure 3F,G). Furthermore, the process of cell senescence, as indicated by the detection of SA-β-gal staining, was observed to be decreased through the administration of ARE treatment (Figure 3H,I). ARE treatment also increased the differentiation potential of PD-MSCs. Adipogenic, ostegenic, and chondrogenic lineages were determined by the staining with Oil Red O, von Kossa, and Alcian blue, respectively, and the mRNA expression levels of their specific markers, *CFD* (Adipsin), *BGLAP* (Osteocalcin), and *COL2A1* (Collagen II), were also increased in ARE-treated PD-MSC compared to untreated cells (Figure 3J). However, ARE treatment had no effect on typical MSC phenotypes, which included expressing markers such as THY1 (CD90), ENG (CD105), and HLA-ABC while lacking CD34, PTPRC (CD45), and HLA-DR (Figure 3K). These findings suggest that ARE increased PD-MSC cell proliferation and differentiation as a functional enhancement.

### 3.3. ARE Increased Antioxidant Factors and Modulate Inflammatory Response in ARPE19 Cell

**Figure 4:**
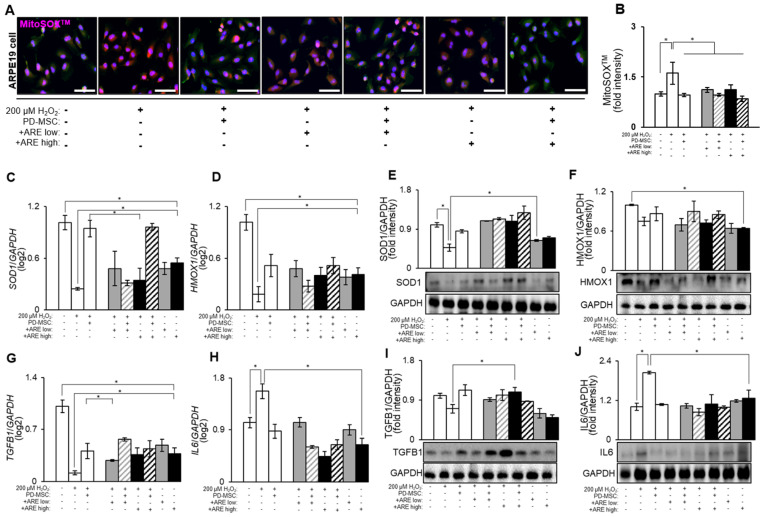
ARE-primed PD-MSCs increased antioxidant and anti-inflammatory effects in ARPE19 cells. (**A**) Oxidative stress levels evaluated in ARPE19 cells using MitoSOX/MitoTracker staining and (**B**) intensity quantified by image J. The gray bar, gray hatch bar, black bar, black hatch bar means ARPE19 cells with H_2_O_2_ and ARE low, ARPE19 cells with with H_2_O_2,_ ARE low and PD-MSCs, ARPE19 cells with H_2_O_2_ and ARE high and ARPE19 cells with with H_2_O_2,_ ARE high and PD-MSCs. The mRNA levels of (**C**) *SOD1* and (**D**) *HMOX1* analyzed by qRT-PCR. The protein levels of (**E**) SOD1 and (**F**) HMOX1 analyzed by Western blot. The mRNA levels of (**G**) *TGFB1*, (**H**) *IL6* analyzed by qRT-PCR. The protein levels of (**I**) TGFB1 and (**J**) IL6 analyzed by Western blot. Scale bar = 200 μm. Each experiment was replicated three times, resulting in a total sample size of three (*n* = 3). The data represent the mean ± SEM. Statistical significance was determined by using one-way ANOVA and Tukey’s post hoc test for the comparison of groups, * *p* < 0.05.

RPE (retinal pigment epithelium) cells are highly metabolically active and are constantly exposed to oxidative stress due to factors like light exposure and high oxygen tension. As a result, they rely on a robust antioxidant defense system to protect themselves from oxidative damage. Antioxidants play a crucial role in maintaining the health and function of RPE cells by neutralizing reactive oxygen species (ROS) and reducing oxidative stress [7,52]. According to the data presented in Figure 4A,B, it can be observed that the application of H_2_O_2_ treatment resulted in a substantial increase in mitochondrial ROS levels in the ARPE19, a human RPE cell line. This increase was measured using the MitoSOX^TM^ indicator and quantified using image J software. Conversely, the administration of high-dose -ARE-primed PD-MSC treatment led to a significant reduction in MitoSOX^TM^ signals, indicating a decrease in oxidative stress. Furthermore, the H_2_O_2_ treatment resulted in a significant decrease in the expression of antioxidant factors such as SOD1 and HMOX1 (HO-1). Conversely, ARE-primed PD-MSC treatment led to dramatically elevating antioxidant levels in the high-dose ARE-primed PD-MSC group (Figure 4C–F). Also, we confirmed the presence of inflammatory markers in ARPE19 cells. The anti-inflammatory marker TGFB1 decreased in the H_2_O_2_ group but increased in the -ARE-primed PD-MSC group. Interestingly, TGFB1 levels were high in the low-dose -ARE-primed PD-MSC treatment group. The expression of the pro-inflammatory marker IL-6 was found to decrease in the PD-MSC treatment group (Figure 4G–J). These data provide evidence that the treatment of ARE resulted in a reduction of oxidative and inflammatory stress in retina cells.

### 3.4. ARE-Primed PD-MSC Increased RPE65 and RDN11 in ARPE19 Cell 

**Figure 5:**
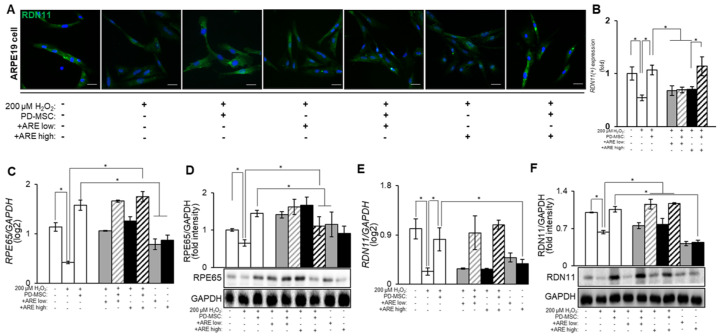
ARE-primed PD-MSC increased RPE65 and RDN11. (**A**) The localization of RDN11 analyzed by IF staining. (**B**) The RDN11 expression quantified by image J program. The gray bar, gray hatch bar, black bar, black hatch bar means ARPE19 cells with H_2_O_2_ and ARE low, ARPE19 cells with with H_2_O_2,_ ARE low and PD-MSCs, ARPE19 cells with H_2_O_2_ and ARE high and ARPE19 cells with with H_2_O_2,_ ARE high and PD-MSCs. The mRNA levels of (**C**) *RPE65* and (**E**) *RDN11* analyzed by qRT-PCR. The protein levels of (**D**) RPE65 and (**F**) RDN11 analyzed by Western blot. Scale bar = 200 μm. Each experiment was replicated 5 times, resulting in a total sample size of 5 (*n* = 5). The data represent the mean ± SEM. Statistical significance was determined by using one-way ANOVA and Tukey’s post hoc test for the comparison of groups, * *p* < 0.05.

The visual cycle comprises to a series of biochemical reactions occurring within the retina of the eye, specifically within the RPE and photoreceptor cells, aimed at regenerating visual pigments necessary for sight. Several pivotal genes and enzymes participate in this process. For instance, the RPE65 gene encodes retinoid isomerase, crucial for converting all-trans-retinyl esters inti 11-cis-retinol during the visual cycle. Additionally, RDN11, a retinol dehydrogenase, participates in this cycle by catalyzing the conversion of 11-cis-retinol to 11-cis retinaldehyde. These gene and enzymes play crucial roles in maintaining the visual cycle by converting retinoids into various forms necessary for regenerating visual pigments in photoreceptor cells [53]. Dysfunction or mutations in these genes can lead to various retinal diseases and vision impairments. We evaluated the protein expression levels of RPE-specific markers, particularly RDN11, in cells using immunostaining with a specific antibody [54]. The application of high-dose -ARE-primed PD-MSC treatment led to the restoration of reduced RDN11 levels (Figure 5A,B). Further, the mRNA levels of RPE65 and RDN11 increased in the -ARE-primed PD-MSC group, particularly in the high-dose primed PD-MSC group (Figure 5C,E). The protein expression of RPE65 and RDN11 also increased in high-dose -ARE-primed PD-MSC treatment group (Figure 5D,F). The results indicate that ARE priming of PD-MSCs upregulated visual cycle-related genes and proteins, particularly with a high -dose of ARE priming of PD-MSCs.

### 3.5. ARE-Activated PI3K/Akt Signaling Pathway in Dry Eye Model

**Figure 6:**
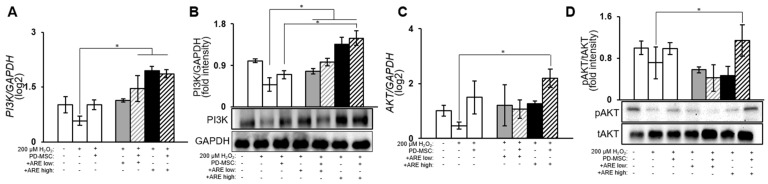
ARE-primed PD-MSC increased PI3K/AKT signaling. The mRNA levels of (**A**) *PI3K*, (**C**) *AKT* were analyzed by qRT-PCR. The protein levels of (**B**) PI3K, (**D**) AKT were analyzed by Western blot. We replicate each experiment triple and total sample amount is 5. Data represent the mean ± SEM. Statistical significance was determined by using one-way ANOVA and Tukey’s post hoc test for the comparison of groups, * *p* < 0.05.

To conduct a more comprehensive examination of the augmented therapeutic effects of PD-MSC with ARE treatment in a retinal injury model, we assessed the protein expression level of the PI3K/AKT signaling pathway. The PI3K/AKT signaling pathway has the capacity to act as a neuroprotective pathway in response to induced retinal injury [55]. It is established that the phosphorylation of AKT serves as a reliable marker for the activation of the PI3K/AKT signaling pathway. The ARE-treated groups exhibited elevated levels of PI3K in comparison to the untreated groups (Figure 6A,B). However, the group treated with high dose ARE PD-MSCs exhibited elevated levels of the active form of AKT in comparison to the untreated groups (Figure 6C,D). The data presented in this study demonstrate that the activation of ARE-primed PD-MSCs resulted in the initiation of the PI3K/AKT signaling pathway within the injured retinal cells.

## 4. Discussion

The human eye comprises diverse cell types, from the corneal epithelium in the anterior chamber to the retinal ganglion cells projecting into the brain. Disruptions to these cells, stemming from pathogenic mutations or environmental factors, can lead to vision loss, susceptible to various disorders from infections and significantly impacting patients’ quality of life. Innovative treatments are essential to prevent progression and restore vision in both inherited and non-genetic ocular disorders. Oxidative stress is a key pathogenic factor in AMD, DR, proliferative vitreoretinopathy, retinopathy, and glaucoma, all conditions affecting the human eye. The retina, being a highly metabolic tissue with significant ROS generation in mitochondria, is at an increased risk of age-associated processes contributing to heightened oxidative stress and neurodegeneration. Damage to retinal cell layers can result in deficits in visual acuity, progressive vision loss, and a significant impact on mobility and quality of life [56]. Enzymatic and nonenzymatic antioxidants form defensive systems against oxidative stress in ocular tissues. Given the crucial role of oxidative stress in AMD pathogenesis and the limited treatment options for non-neovascular dry AMD, therapeutic strategies focused on oxidative stress are promising as a potential addition to established methods.

Understanding the various mechanisms through which natural compounds protect visual function is a highly complex process. Although numerous natural substances are proposed for various types of age-related macular degeneration (AMD), screening and validating these compounds requires extensive effort and research.

The present study revealed that the application of ARE treatment resulted in an augmentation of cell proliferation, increased expression of stemness markers, and enhanced differentiation potential of PD-MSCs. Moreover, the application of ARE treatment on an injured human retinal ARPE19 cell line with H_2_O_2_ treatment demonstrated the reinstatement of retinal cells and the restoration of antioxidant and anti-inflammatory signals of PD-MSCs. Furthermore, we found that the upregulating of visual cycle related gene was achieved through ARE-primed PD-MSCs, effectively reactivating the PI3K/Akt signaling pathway. This discovery represents the first instance of functionally enhanced MSCs stimulating the antioxidant defense system in human eye cells, facilitating recovery from chemical damage (Figure 6). The paracrine effects of MSCs offer a promising avenue for enhancing eye function, with their secretion of growth factor, cytokines, and bioactive molecules facilitating in tissue restoration within the eye. This therapeutic potential has led to investigations into MSC priming, a strategy involving pre-treatment or genetic manipulation to augment their paracrine effects further [32,57]. Studies suggest that priming MSCs can increase their secretion of soluble factors, contributing to improved survival and functionality of ocular cells [58]. Our study found that the response of PD-MSC to ARE shows a tendency dependent on the concentration of the substance. The expected enhancement of the antioxidant effect, anti-senescence, and anti-inflammatory effect did occur in the presence of a high concentration of ARE. However, in some cases, low ARE dose treatment had more effect on ARPE19 cells (Figure 3I). The reason for natural substances’ better expression at low concentrations can be explained by various physiological and biochemical factors. One of these is cell sensitivity. Cells can experience stress when exposed to high concentrations of natural substances. At low concentrations, cells can avoid this stress while benefiting from the positive effects of the natural substances [59]. Another factor is toxic effects. High concentrations of natural substances can be toxic to cells [60]. A third factor is cell signaling pathways. When natural substances activate specific cell signaling pathways, low concentrations can optimally activate these pathways [61]. Additionally, according to the supplementary data, when ARE alone was applied to ARPE-19 cells, they showed lower gene expression compared to the ARE + -PD-MSC group. Collectedly, our data indicated that ARE has a more positive effect when primed with stem cells compared to its effect on the cells alone. This suggests that priming with stem cells enhances the function of cell. These findings imply that a combination of ARE with PD-MSCs could potentially be a more effective therapeutic strategy. From these data, we conclude that high-dose ARE might more effective. Further studies are planned to be conducted both in vitro and in vivo based on these findings. However, the limitation of natural compound mixtures or extracts, which are unidentified and complex, may contain unknown factors that exert distinct effects on cellular metabolism. Therefore, to enhance the biological utilization of natural compounds, the following research efforts are necessary: enhancement of absorption, pharmacological characterization, molecular design and optimization and in vivo distribution studies. These research efforts provide opportunities to more effectively apply natural compounds, playing a crucial role in protecting and restoring visual function. 

Future research should explore the dependency of concentration or the optimal concentration of the main active compound, as well as the potential inhibitory effects of other factors. The analysis of natural products is challenging due to complex mixtures, diverse structures, and low concentrations. Challenges involve matrix interference, difficulties in isolation, and stability concerns. The absence of reference standards and biological variability adds complexity. 

**Figure 7:**
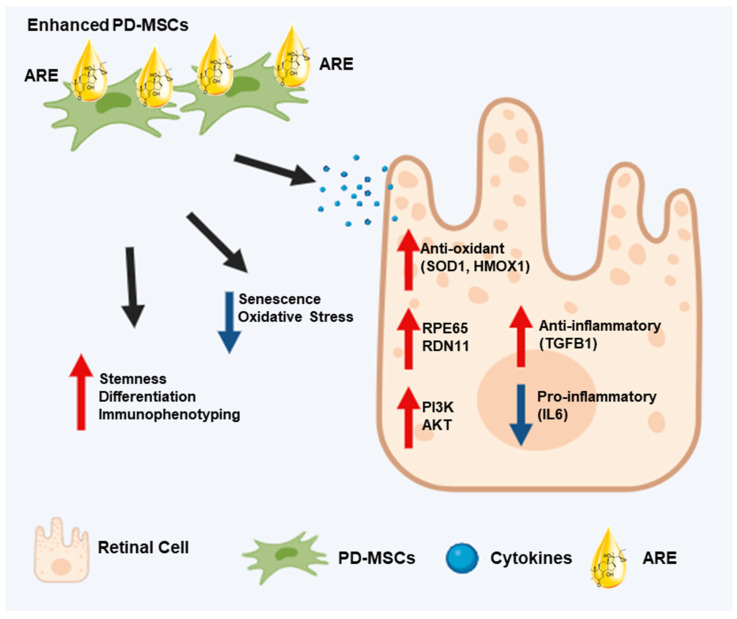
Effects of ARE on therapeutic potential of PD-MSCs on chemically-injured retinal cells. The therapeutic efficacy of ARE-primed PD-MSCs is (1) inhibiting inflammation in injured retinal cells, (2) activating PI3K/AKT signaling, (3) reducing oxidative stress by increasing the expression of antioxidant factors, and (4) promoting the regeneration of injured retinal cells by increasing the expression of regeneration-related factors.

In the context of managing ocular diseases like non-neovascular dry AMD, antioxidant medications utilizing PD-MSCs functionally enhanced by ARE priming are poised to play a crucial role in therapeutic interventions. The exploration of MSC priming to amplify both antioxidant capabilities and paracrine effects holds promise, especially for achieving specific antioxidant effects in retinal disorders. Nonetheless, further research is needed to explicitly establish the connection between antioxidants and the repair of injured eyes. This novel approach holds potential for advancing cellular treatments in ocular diseases (Figure 7). 

## 5. Conclusions

This study explored the enhanced functionalities of PD-MSCs through the augmentation induced by ARE. Notably, this enhancement manifested in the heightened expression of genes related to antioxidants, anti-inflammation, and improved eye function, driven by the activation of the PI3K/AKT signaling pathway. The findings strongly advocate for the potential of employing these functionally enhanced PD-MSCs as a cutting-edge strategy in the evolution of stem cell therapy for treating various ocular diseases. This promising avenue could significantly shape the landscape of advanced treatments for ocular disorders in the future.

## Figures and Tables

**Table 1:** Primer sequences using quantitative real-time polymerase chain reaction.

Gene	Primer	Annealing Temperature (°C)
*POU5F1* (*OCT4*)	F: 5′-AGTGAGAGGCAACCTGGAGA-3′	52
	R: 5′-GTGAAGTGAGGGCTCCCATA-3′	
*NANOG*	F: 5′-TTCTTGACTGGGACCTTGTC-3′	52
	R: 5′-GCTTGCCTTGCTTTGAAGCA-3′	
*TERT*	F: 5′-GAGCTGACGTGGAAGATGAG-3′	55
	R: 5′-CTTCAAGTGCTGTCTGATTCCAATG-3′	
*HLA-G*	F: 5′-GCGGCTACTACAACCAGAGC-3′	58
	R: 5′-GCACATGGCACGTGTATCTC-3′	
*ACTB* (*β-actin*)	F: 5′-TCCTTCTGCATCCTGTCAGCA-3′	58
	R: 5′-CAGGAGATGGCCACTGCCGCA-3′	
*CFD* (*Adipsin*)	F: 5′-GGTCACCCAAGCAACAAAGT-3′	57.8
	R: 5′-CCTCCTGCGTTCAAGTCATC-3′	
*BGLAP* (*Osteocalcin*)	F: 5′-TGAATCGGAACAACCTGACTGA-3′	59.1
	R: 5′-TTCCACTAGCAAGAAGAAGCCTTT-3′	
*COL2A1* (*Col II*)	F: 5′-CTCGTGGCAGAGATGGAGAA-3′	58
	R: 5′-CACCAGGTTCACCAGGATTG-3′	
*RPE65*	F: 5′-CGCCAGAACCGTGCAGA-3′	59.1
	R: 5′-TCAGGCTGCTGGCTGAC-3′	
*RDN11*	F: 5′-AGCAGGTGTTGGTGCGGAAACT-3′	62
	R: 5′-CGGACACATCATCACTCCTGCA-3′	
*SOD1*	F: 5′-GCTGTACCAGTGCAGGTCCTCA-3′	60
	R: 5′-CATTTCCACCTTTGCCCAAGTC-3′	
*HMOX1* (*HO-1*)	F: 5′-GCGAAACAAGCAGAACCCA-3′	58.5
	R: 5′-GCGAAACAAGCAGAACCCA-3′	
*TGFB1*	F: 5′-TACCAGAAATACAGCAACAATTCC-3′	57
	R: 5′-AAAGCCCTCAATTTCCCCTCC-3′	
*IL6*	F: 5′-TCCACAAGCGCCTTCGGTCCAGTTG-3′	67
	R: 5′-AGAGGTGAGTGGCTGTCTGTGTGGG-3′	
*PI3K*	F: 5′-GGAGCCTGGAAGAGCCC-3′	57.8
	R: 5′-CGTGGAGGCATTGTTCTGAT-3′	
*AKT*	F: 5′-GTCGCCTGCCCTTCTACAAC-3′	59.5
	R: 5′-CACACGATACCGGCAAAGAA-3′	
*GAPDH*	F: 5′-GCACCGTCAAGGCTGAGAAC-3′	60
	R: 5′-GTGGTGAAGACGCCAGTGGA-3′	

## Data Availability

The data presented in this study are available on request from the corresponding author. The data are not publicly available due to privacy/ethical restrictions.

## Supplementary Materials

The following supporting information can be downloaded at: https://www.mdpi.com/article/10.3390/cells13141229/s1, Figure S1: The MTT assay results and effects of indicated concentrations of Ecdysyerone; Figure S2: The MTT assay results and effects of indicated concentrations of ARE on the cell viability are presented.
