# Supplementary material for: Achyranthis radix Extract Enhances Antioxidant Effect of Placenta-Derived Mesenchymal Stem Cell on Injured Human Ocular Cells
Source: Cells. 2024 Jul 21;13(14):1229. doi: 10.3390/cells13141229 (PMC11274440; doi:10.3390/cells13141229)
Supplement: Supplementary file 1 [file cells-13-01229-s001.zip › cells-3091231-supplementary.pdf]

Supplementary materials

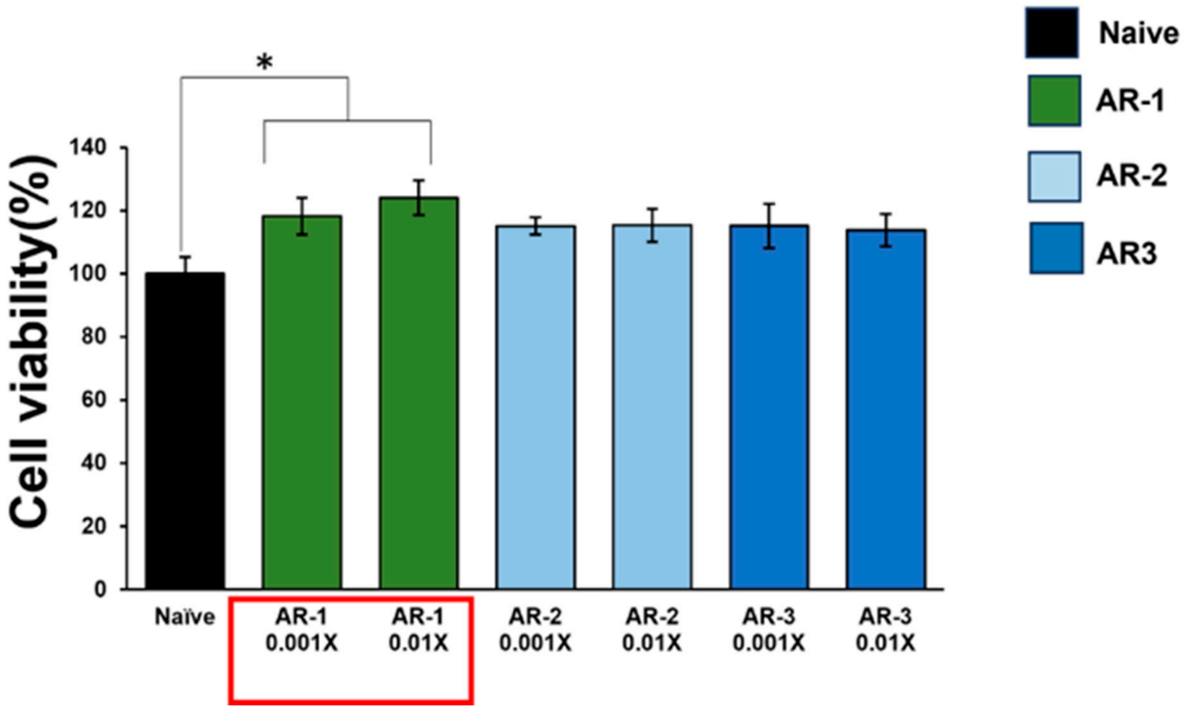

AR-1	Ecdysterone	5289-74-7	C27H44O7 (480.6)
AR-2	25R-Inokosterone	19682-38-3	C27H44O7 (480.6)
AR-3	25S-Inokosterone	19595-18-7	C27H44O7 (480.6)

Figure S1. The MTT assay results and effects of indicated concentrations of Ecdysyerone, 25R-Inokosterone and 25S-Inokosterone on the cell viability are presented. The data represent the mean ± SEM. Statistical significance was determined by using one-way ANOVA and Tukey's post hoc test for the comparison of groups, *$p < 0.05$, vs Naive.

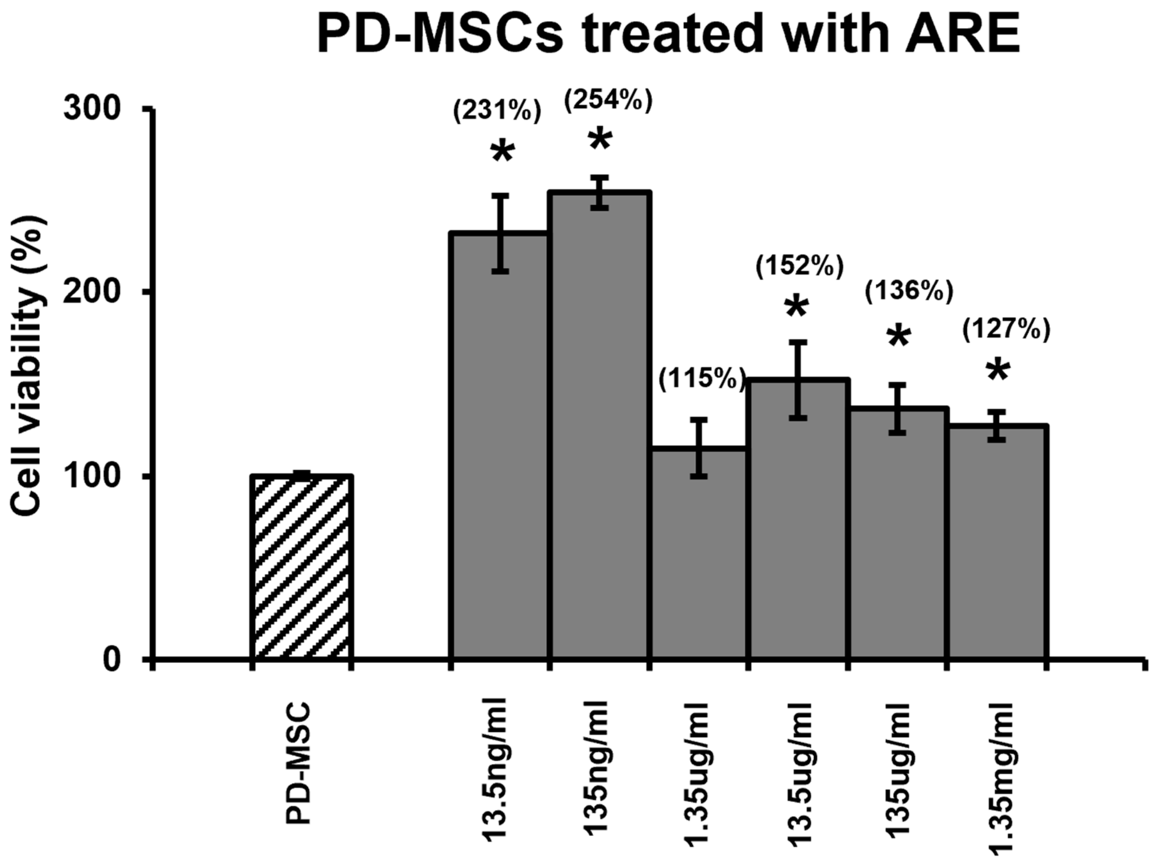

Figure S2. The MTT assay results and effects of indicated concentrations of ARE on the cell viability are presented. The data represent the mean ± SEM. Statistical significance was determined by using one-way ANOVA and Tukey's post hoc test for the comparison of groups, *p< 0.05, vs PD-MSC.
